# Von willebrand factor increases endothelial cell adhesiveness for human mesenchymal stem cells by activating p38 mitogen-activated protein kinase

**DOI:** 10.1186/scrt35

**Published:** 2010-11-17

**Authors:** Irina A Potapova, Ira S Cohen, Sergey V Doronin

**Affiliations:** 1 Department of Physiology and Biophysics, Stony Brook University, Nicolls Road, Stony Brook, NY 11794, USA

## Abstract

**Introduction:**

Delivered systemically or natively circulating mesenchymal stem cells accumulate in injured tissues. During homing mesenchymal stem cells adhere to endothelial cells and infiltrate underlying tissue. Previously we have shown that adhesiveness of endothelial cells for mesenchymal stem cells correlates with the inhibition of mitochondrial function of endothelial cells and secretion of von Willebrand factor. We hypothesized that von Willebrand factor is an auto/paracrine regulator of endothelial cell adhesiveness and studied the effect of von Willebrand factor on adhesion of mesenchymal stem cells to endothelial cells.

**Methods:**

We used Affymetrix DNA microarrays, human protein phospho-MAPK array, Western blot, cell-based ELISA and flow cytometry analysis to study the activation of endothelial cells by von Willebrand factor. Cell adhesion assay and protein kinase inhibitors were used to evaluate the role of mitogen-activated protein kinases in the regulation of endothelial cell adhesiveness for mesenchymal stem cell.

**Results:**

Treatment of endothelial cells with von Willebrand factor stimulated the mesenchymal stem cell adhesion in a time- and concentration-dependent manner. Mesenchymal stem cells did not adhere to immobilized von Willebrand factor and did not express receptors for von Willebrand factor suggesting that the stimulation of the mesenchymal stem cell adhesion is a result of endothelial cell activation with von Willebrand factor. Treatment of endothelial cells with von Willebrand factor activated ERK-1,2 and p38 MAPK without an effect on gene or cell surface expression of E-selectin, P-selectin, VCAM1 and ICAM1. Inhibition of p38 MAPK, but not ERK-1,2, in endothelial cells completely abrogated the stimulation of the mesenchymal stem cell adhesion by von Willebrand factor.

**Conclusions:**

Von Willebrand factor is an auto/paracrine regulator of endothelial cells. Activation of p38 MAPK in endothelial cells by von Willebrand factor is responsible for the regulation of endothelial cell adhesiveness for mesenchymal stem cells.

## Introduction

Systemically delivered or natively circulated mesenchymal stem cells (MSCs) target tissues affected by radiation, infarction and other kinds of trauma [1-4]. During the homing MSCs are likely to utilize multiple mechanisms for recognition of injured tissues. One such mechanism may include adhesion of MSCs to distressed/apoptotic endothelial cells (ECs). ECs show limited adhesiveness for cells circulating in the bloodstream, however, they became activated after exposure to inflammatory or stress factors. Activation of ECs under stress conditions occurs rapidly and results in massive release of von Willebrand factor (vWF) from intracellular storage. Immobilization of vWF on the surface of ECs and an extracellular matrix causes platelet adhesion and aggregation. Recent studies have shown that endothelial stress may also play a significant role in the regulation of stem cell homing [5]. Previously we have shown that adhesion of human mesenchymal stem cells (hMSCs) to ECs *in vitro *is regulated by endothelial distress and apoptosis and correlates with the inhibition of mitochondrial function in ECs and the release of vWF [6]. In this study we demonstrate that vWF stimulates p38 MAPK that regulates EC adhesiveness for hMSCs.

## Materials and methods

### Reagents

Human vWF-Factor VIII free was obtained from American Diagnostica Inc. (Stamford, CT, USA). P38 MAPK and ERK-1,2 inhibitors, 4-(4-fluorophenyl)-2-(4-methylsulfinylphenyl)-5-(4'-pyridyl)-1-H-imidasole (SB203580), 4-ethyl-2(p-methoxyphenyl)-5-(4'-pyridyl)-1-H-imidazole (SB202474), 2'-amino-3'-methoxyflavone (PD98059), 1,4-diamino-2,3-dicyano-1,4-bis(2-aminophenylthio)butadiene (U0126), were purchased from Calbiochem (Gibbstown, NJ, USA). Neutralizing antibodies against human P-selectin, E-selectin, ICAM1, VCAM1 and normal IgG (isotype-matching control) were purchased from R&D Systems (Minneapolis, MN, USA).

### Cell culture

Human mesenchymal stem cells (hMSCs) and human umbilical vein endothelial cells (HUVECs) were purchased from Lonza Group Ltd. (Basel, Switzerland) and cultured in MSCGM BulletKit (Lonza) and EGM-2 BulletKit (Lonza), accordingly. Passages 2 to 5 were used. Cells were maintained at 37°C in a humidified atmosphere of 5% CO_2_.

### HMSC adhesion assay

HMSC adhesion to HUVECs was conducted as previously described [6]. HMSCs grown as a monolayer were dissociated with trypsin-EDTA solution (Lonza), washed with Hank's balanced salt solution (HBSS), and labeled with 4 μg/ml calcein AM (Molecular Probes, Invitrogen, Carlsbad, CA, USA) in HBSS for 45 minutes at 37°C and 5% CO_2_. After the labeling, hMSCs were washed with HBSS and resuspended in Dulbecco's modified Eagle's medium (DMEM; Sigma, St. Louis, MO, USA). HUVECs were prepared for the adhesion assay as follows. A confluent monolayer of HUVECs in a 96-well cell culture clear-bottom black plate (Corning Incorporated Life Sciences, Lowell, MA, USA) was washed twice with HBSS and treated with vWF (0 to 6 μg/ml) in HBSS for 0 to 9 hours at 37°C and 5% CO_2_.

Before the adhesion assay cells were washed with HBSS and left in 50 μl of HBSS. HMSC suspension (50 μl, 10,000 cells per well) was added to HUVECs and incubated for 30 minutes at 37°C and 5% CO_2_. The cell load was estimated by scanning the plate in a POLARstar OPTIMA microplate reader (BMG Labtech Inc., Cary, NC, USA) at excitation/emission wavelengths of 485/520 nm. Wells without hMSCs were used to assay the background fluorescence. Unbound hMSCs were aspirated and wells were washed with 200 μl of HBSS two times, 100 μl HBSS was added to each well and plates were scanned to assay a number of bound cells. The percentage of bound cells was calculated as a ratio between the fluorescence of washed and unwashed wells after subtraction of the background fluorescence from both values. At least six wells were used for each experimental condition. At least three independent experiments were conducted for each treatment.

Adhesion of hMSCs to collagen-coated or tissue culture plates was studied using a 96-well collagen I coated clear-bottom black plate (BD Biosciences, Franklin Lakes, NJ, USA) and a 96-well cell culture clear-bottom black plate, respectively. Immobilization of vWF was achieved by incubation of plates with a solution of vWF (0 to 8 μg/ml) in HBSS for four hours. The adhesion of hMSCs to the plates was assayed before and after vWF immobilization. In order to remove unbound vWF wells were washed with HBSS before the adhesion assay. Immobilization of vWF on collagen I coated and tissue culture plates was monitored by ELISA. Plates were treated with vWF as described above, washed three times with the wash buffer from ELISA development kit (R&D Systems) and incubated with peroxidase-conjugated rabbit polyclonal anti-human vWF antibody (Dako North America, Inc., Carpinteria, CA, USA) according to the manufacturer's recommendations. ELISA was developed and the optical densities were measured at 450 nm with a 595 nm reference wavelength in a POLARstar OPTIMA microplate reader (BMG Labtech Inc., Cary, NC, USA). Immobilization of vWF was determined using eight measurements per each experimental condition.

In experiments on the inhibition of p38 MAPK and ERK-1,2 HUVECs were pre-incubated with inhibitors of protein kinases for 45 minutes and then stimulated with vWF. Activity of p38 MAPK was inhibited with SB203580. SB202474, a chemical analog of SB203580, was used as a negative control. Phosphorylation and activation of ERK-1,2 was inhibited with U0126 or PD98059.

### Flow cytometric analysis of antigen expression on the surface of hMSCs and HUVECs

Analysis of E-selectin, VCAM1 and ICAM1 expression on the surface of HUVECs and integrin α_v_β_3 _and GPIbα expression on the surface of hMSCs was conducted using flow cytometry. Cells were dissociated and resuspended in the flow cytometry buffer consisting of 2% bovine serum albumin (Sigma) and 0.1% sodium azide (Sigma-Aldrich, St. Louis, MO, USA) in Dulbecco's phosphate buffered saline (PBS; Sigma). HUVECs were dissociated using Hank's based enzyme free cell dissociation solution (Millipore, Billerica, MA, USA). HMSCs were dissociated with trypsin-EDTA solution (Lonza). Cells (2 × 10^5 ^cells) were stained with corresponding fluorochrome-conjugated monoclonal antibodies (BD Biosciences) for 30 minutes at room temperature according to the manufacturer's recommendations. After incubation with antibodies, cells were washed with 5 ml of the flow cytometry buffer and resuspended in the flow cytometry buffer containing 1% paraformaldehyde (Electron Microscopy Sciences, Hatfield, PA, USA). Background staining was assessed by incubation of cells with mouse fluorochrome- and isotype-matching immunoglobulins. Flow cytometric analysis was performed by acquiring 5,000 events on a FACScan flow cytometer (BD Biosciences, Franklin Lakes, NJ, USA). Data were processed with a CellQuest™software package supplied by instrument manufacturer (BD Biosciences, Franklin Lakes, NJ, USA). The cellular debris was assessed on the basis of forward and right angle scattering analysis and excluded from further analysis by a CellQuest™software package (BD Biosciences, Franklin Lakes, NJ, USA).

### Human phospho-MAPK array

Analysis of protein kinase phosphorylation in HUVECs treated with vWF was conducted using the human phospho-MAPK array kit (R&D Systems). Confluent HUVECs grown on a 100 mm tissue culture plate were washed twice with HBSS and treated with 4 μg/ml vWF in HBSS for 0 to 35 minutes. After the treatment cells were washed with HBSS and lysed with the manufacturer supplied buffer and protein, phosphorylation was developed according to the manufacturer's recommendations. Phosphorylation of protein kinases was detected by exposure of phospho-MAPK array to X-ray film (Kodak, Rochester, NY, USA). All arrays from the same experiment were processed simultaneously and exposed to the same X-ray film.

### Western blot analysis of p38 MAPK and ERK-1,2 phosphorylation

Confluent HUVECs grown on a 100 mm tissue culture plate were washed twice with HBSS, treated with 4 μg/ml vWF in HBSS for 0 to 5 minutes and lysed with 1 ml of lysis buffer containing 0.025 M Tris-HCl, pH 7.4, 0.15 M NaCl, 5 mM EDTA, 1% Triton X-100, 0.5% Nonidet P-40, and a set of protease inhibitors (Roche Applied Science, Indianapolis, IN, USA) and phosphatase inhibitors (cocktails type 1 and 2) (Sigma) for 15 minutes at 4°C. Extract was cleared by centrifugation at 15,000 × *g *at 4°C for 30 minutes. Proteins (25 μg) were separated in Bis-Tris 10% Criterion gel (Bio-Rad, Hercules, CA, USA) using XT MOPS running buffer (Bio-Rad) and transferred to a nitrocellulose membrane (Bio-Rad). Western blot was performed using polyclonal antibodies against phosphorylated p38 MAPK (Thr180/Tyr182), total p38 MAPK, phosphorylated ERK-1,2 (Thr202/Tyr204) and total ERK-1,2 (Cell Signaling Technology, Danvers, MA, USA). Western blot was developed with Rabbit TrueBlot HRP-labeled anti-rabbit antibody (eBioscience, San Diego, CA, USA) and ECL Western blotting detection reagents (GE Healthcare UK Limited, Pittsburgh, PA, USA).

### Cell-based ELISA for p38 MAPK and ERK-1,2 phosphorylation in HUVECs treated with vWF

Phosphorylation of p38 MAPK (Thr180/Tyr182) and ERK-1,2 (Thr202/Tyr204) was assayed using corresponding cell-base ELISA kits (R&D Systems). Confluent HUVECs grown on a 96-well cell culture clear-bottom black plate were washed twice with HBSS and treated with 0 to 6 μg/ml vWF in HBSS for four hours. After the treatment, cells were washed with HBSS, fixed with 4% paraformaldehyde in phosphate-buffered saline for 30 minutes and stained according to the manufacturer's recommendations. Fluorescence of total protein kinase at 450 nm and phosphorylated protein kinase at 600 nm were acquired in a POLARstar OPTIMA microplate reader. Background fluorescence was estimated as recommended by the manufacturer from the control wells stained with corresponding secondary antibody and the relative ratio of the fluorescence of phosphorylated protein kinase to the fluorescence of total protein kinase was calculated. At least six wells were used for each experimental condition.

### Activity assays of p38 MAPK and ERK-1,2

Activities of p38 MAPK and ERK-1,2 in HUVEC lysate were assayed using the p38 and p44/42 MAPK assay kits (Cell Signaling Technology). Confluent HUVECs grown on a six-well cell culture plate were washed twice with HBSS and treated with 0 to 6 μg/ml vWF in HBSS for four hours. After the treatment cells were washed with HBSS and lysed with the provided buffer according to the manufacturer's recommendations. Phosphorylated p38 MAPK (Thr180/Tyr182) and ERK-1,2 (Thr202/Tyr204) were immunoprecipitated from HUVEC lysates of equal volume (1 ml) and protein concentrations (1.5 mg/ml) with corresponding anti-phospho-p38 MAPK (Thr180/Tyr182) and anti-phospho ERK-1,2 (Thr202/Tyr204) antibodies supplied by the manufacturer. Enzymatic activities of immunoprecipitated protein kinases were assayed using recombinant ATF-2 protein as a substrate for p38 MAPK and recombinant Elk-1 protein as a substrate for ERK-1,2. Phosphorylation of ATF-2 and Elk-1 proteins was detected by Western blots. For this, reaction mixtures (35 μl) were separated in Bis-Tris 10% Criterion gel using XT MOPS running buffer and transferred to a nitrocellulose mem-brane. Western blot was performed using anti-phospho-ATF-2 (Thr71) or anti-phospho-Elk-1 (Ser383) antibodies. Immunoreactive bands were visualized using affinity purified HRP-labeled goat anti-rabbit F(ab')2 fragment antibody (Kirkegaard and Perry Laboratories, Gaithersburg, Maryland, USA) and ECL Western blotting detection reagents (GE Healthcare UK Limited).

### Affymetrix DNA microarray analysis

RNA was extracted from HUVECs using the RNeasy kit (Qiagen, Germantown, MD, USA), and analysis of gene expression in HUVECs was performed on Affymetrix Human Genome U133 Plus 2.0 array (Santa Clara, CA, USA) according to the manufacturer's recommendations. Raw microarray data were processed with the affy package of the Bioconductor project using MAS 5.0 algorithm and subjected to t-test.

### Analysis of DNA content using Quanti-iT PicoGreen dsDNA reagent

HUVECs were treated with vWF and inhibitors of protein kinases as described above and washed with HBSS. To prepare the cell lysate 100 μl of cell lysis solution (0.2% v/v Triton X-100, 10 mM Tris (ph 7.0), 1 mM EDTA) was added to each well (96-well plate), and the plate was process through a total of two freeze at -80°C/thaw at room temperature cycles. After a final thaw 100 μl of the aqueous working solution of Quant-iT PicoGreen dsDNA reagent (Invitrogen) prepared according the manufacturer's instructions was added to each well. Fluorescence was measured using a Polarstar OPTIMA microplate reader (BMG Labtech Inc.) at excitation/emission wavelengths of 485/520 nm. DNA standard curve was generated using dsDNA standard provided with the Picogreen Assay kit and used for determining the DNA concentration of the samples.

### Confocal imaging

Confluent monolayer of HUVECs on Lab-Tek II chamber CC2 glass slides (Nalge Nunc International, Rochester, NY, USA) was treated with 4 μg/ml vWF in HBSS for four hours and the adhesion assay with hMSCs was conducted as described in the hMSC adhesion assay section. Cells were fixed with 5% paraformaldehyde, permeabilized with 0.1% Triton x100 in phosphate buffered saline, blocked with 5 mg/ml bovine serum albumin in PBS for one hour and stained with 1 μg/ml AF488-conjugated CD31 antibody (BD Pharmingen, Franklin Lakes, NJ, USA), the specific antigen marker of HUVECs, and 1 μg/ml PE-conjugated CD90 antibody (BD Pharmingen), the specific antigen marker of hMSCs, for four hours. After staining cells was washed with PBS and images were acquired on Olympus FluoView FV1000 confocal microscope (Olympus America Inc. Center Valley, PA, USA).

## Results

### VWF regulates hMSC adhesion to HUVECs

Previously we have shown that endothelial distress potentiates the hMSC adhesion [6]. The adhesion of hMSCs to distressed/apoptotic HUVECs correlated with the secretion of vWF by ECs suggesting that vWF may regulate the interaction of hMSCs with ECs [6].

In order to study the effect of vWF on the hMSC adhesion HUVECs were treated with exogenous vWF. Treatment of HUVECs with vWF was conducted in HBSS to eliminate the interference from vWF present in fetal bovine serum. Plates were washed before the adhesion assay in order to remove unbound vWF. Since hMSCs respond to endothelial distress [6], and serum deprivation itself is a stress factor, we also tested whether HBSS alone affects the hMSC adhesion.

**Figure 1:**
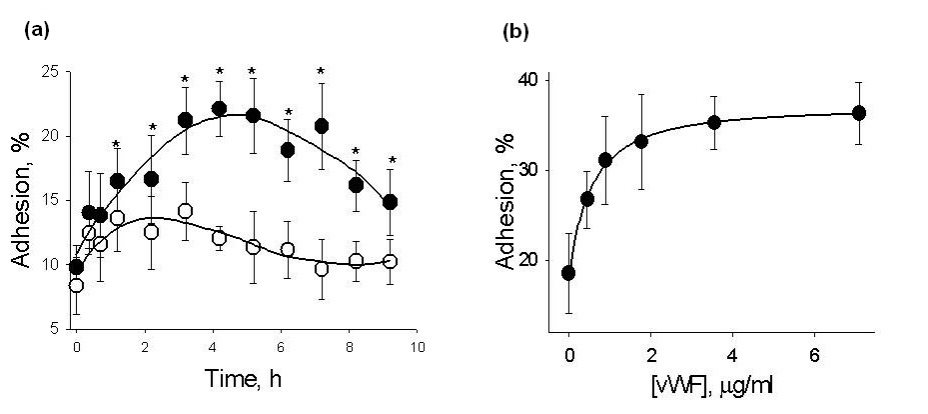
**VWF stimulates HUVEC adhesiveness for hMSCs**. **(a) **Shows changes in HUVEC adhesiveness for hMSCs caused by treatment with 4 μg/ml vWF (black circle) or HBSS (white circle) for 0 to 9 hours. Asterisks mark time points where adhesion of hMSCs to HUVECs treated with vWF was different (t-test, *P*-value <0.05) from the adhesion to HUVECs maintained in HBSS. **(b) **Shows the dose-response curve of HUVEC adhesiveness for hMSCs after treatment of HUVECs with 0 to 6 μg/ml vWF for four hours. Data are shown as mean ± SD of eight independent measurements.

**Figure 2:**
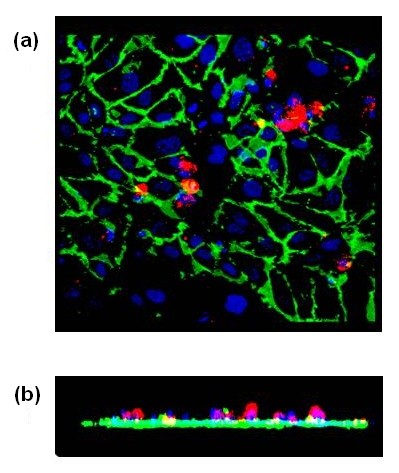
**Image of hMSCs adherent to a confluent monolayer of HUVECs treated with vWF**. Confocal image of a planar **(a) **and Z-axis **(b) **projections of hMSCs adherent to HUVECs treated with 4 μg/ml vWF for four hours. HUVECs were stained with AF488-conjugated CD31 (green). HMSCs were labeled with PE-conjugated CD90 (red). HMSCs were found on the top of endothelial monolayer within the boundaries of ECs.

VWF stimulated hMSC adhesion to HUVECs in a time and dose-dependent manner (Figure 1). Incubation of HUVECs with HBSS for four hours stimulated the hMSC adhesion 1.3-fold (Figure 1a), which was less than the stimulation caused by the treatment with vWF (2.4-fold). Microscopic examination has shown that hMSCs adhere to HUVECs and are located on the top of endothelial monolayer within the boundaries of ECs. Exemplar confocal image of hMSCs adhered to HUVECs treated with vWF is shown in Figure 2. These data argue that vWF regulates hMSC adhesion to ECs.

### HMSCs do not express glycoprotein 1bα, integrin α_V_β_3 _and do not adhere to immobilized vWF

**Figure 3:**
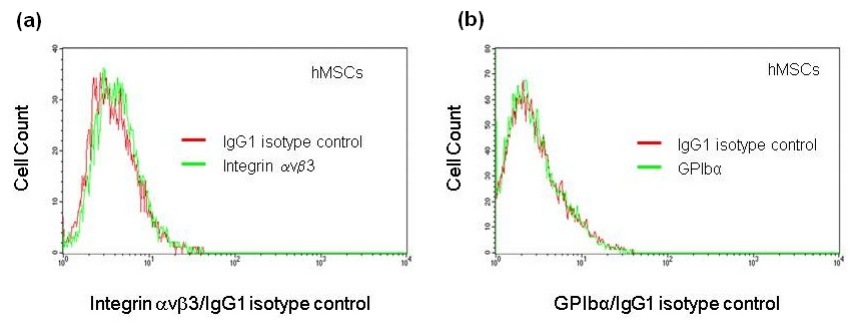
**Expression of α_V_β_3 _integrin and platelet glycoprotein Ibα on the surface of hMSCs**. **(a and b) **Show the results of flow cytometric analysis of integrin α_v_β_3 _((a), green histogram) and platelet glycoprotein Ibα (GPIb, (b), green histogram) expression on the surface of hMSCs. Red histograms represent fluorochrome matching isotype controls.

**Figure 4:**
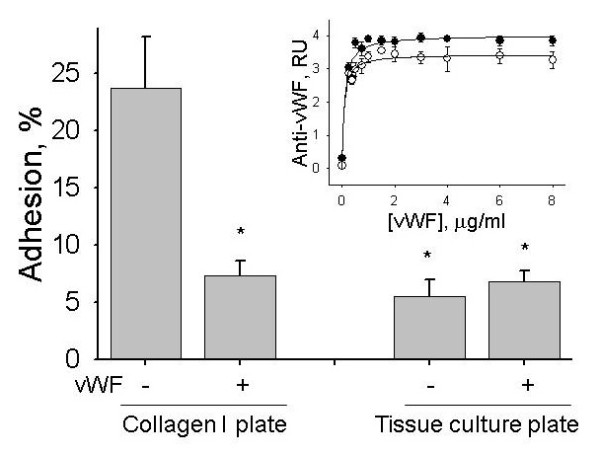
**Effect of vWF on adhesion of hMSCs to collagen I coated or tissue culture treated plastic plates**. Adhesion of hMSCs to collagen I coated and tissue culture plates was measured before and after immobilization of vWF. Before the adhesion assay vWF was removed and plates were washed with HBSS. Asterisks mark statistically significant differences compared to collagen I coated plate (t-test, *P*-value <0.05). Data are shown as mean ± SD of eight independent measurements. The insert shows vWF immobilization on collagen I coated (black circle) and tissue culture (white circle) plates exposed to 0 to 8 μg/ml vWF in HBSS for four hours. Immobilization of vWF was measured by ELISA and is shown in relative units (RU) as mean ± SD of eight independent measurements.

Since the presence of soluble vWF was not required for the stimulation of the MSC adhesion, it was conceivable that the MSC adhesion is regulated by MSC interaction with vWF bound to the endothelial surface or an extracellular matrix. A similar mechanism was suggested for the stimulation of platelet adhesion and aggregation by immobilized vWF [7,8]. Known receptors for vWF include platelet glycoprotein Ib (GPIbα, expressed on the surface of platelets) and α_V_β_3 _integrin (expressed on the surface of platelets and ECs) [7,8]. We, therefore, tested whether hMSCs express receptors for vWF (α_V_β_3 _integrin and GPIbα) and adhere to immobilized vWF. Expression of α_V_β_3 _integrin and GPIbα on the surface of hMSCs was tested by flow cytometric analysis. We found that hMSCs are negative for the expression of integrin α_V_β_3 _and GPIbα (Figure 3). Adhesion of MSCs to vWF was studied after vWF immobilization on tissue culture treated (plastic) or collagen I coated cell culture plates. The insert in Figure 4 shows that nearly equal amounts of vWF were immobilized on plastic or collagen I surfaces after treatment of plates with 0 to 8 μg/ml vWF for four hours. Immobilization of vWF onto plastic plate did not affect hMSC adhesion (Figure 4). HMSCs adhered 3.3 times better to collagen I coated plate than to tissue culture treated plate. Immobilization of vWF on collagen I coated plate inhibited hMSC adhesion (Figure 4) indicating that hMSCs and vWF compete for the binding to collagen I.

Taken together, the data of flow cytometry and the adhesion assay suggest that hMSCs do not directly interact with immobilized vWF, presumably, due to the lack of vWF receptors on the surface of hMSCs.

### Adhesion of hMSCs to HUVECs activated with vWF does not depend on overexpression of the adhesion molecules on the endothelial surface

Lack of direct adhesion of hMSCs to immobilized vWF suggested that the stimulation of the hMSC adhesion is a result of an activation of ECs with vWF. Activation of ECs may occur as a consequence of *de novo *transcription, synthesis and delivery of adhesion molecules (E-selectin, P-selectin, VCAM1 or ICAM1) to the cell surface [9].

In order to evaluate the effect of vWF on gene expression in HUVECs we used Affymetrix DNA microarrays. The set of Affymetrix Human Genome U133 Plus 2.0 arrays (GEO accession (GEO:GSE19816)) included HUVECs maintained in EGM2 growth media (three microarrays), HUVECs treated with HBSS (four hours, three microarrays) and HUVECs treated with vWF (4 μg/ml vWF, four hours, three microarrays). Expression of 340 genes was affected by more than two-fold (*P*-value < = 0.05) in HUVECs treated with HBSS in comparison with HUVECs maintained in EGM2 growth media. Treatment of HUVECs with vWF changed the expression of 157 genes by more than two-fold in comparison with HBSS (Additional file 1) and 567 genes in comparison with EGM2 growth media (Additional file 2). Gene expression of E-selectin, P-selectin, VCAM1 and ICAM1 was not affected by serum starvation or by treatment of HUVECs with vWF.

**Figure 5:**
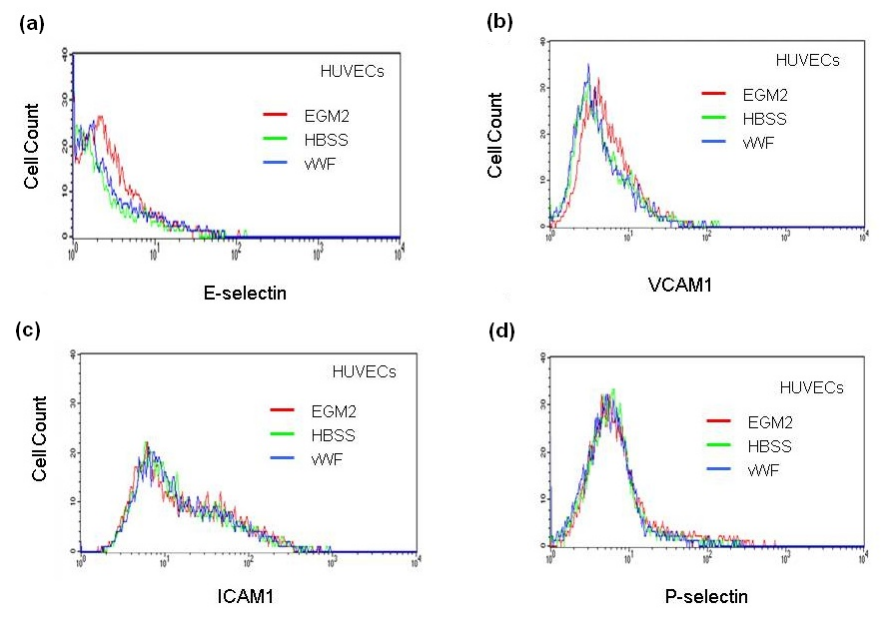
**Expression of E-selectin, P-selectin, VCAM1 and ICAM1 on the surface of HUVECs treated with vWF**. HUVECs were treated with 4 μg/ml vWF in HBSS for four hours. Expression of E-selectin **(a)**, VCAM1 **(b)**, ICAM1 **(c) **and P-selectin **(d) **on the surface of HUVECs maintained in EGM2 growth media (red histogram), incubated with HBSS (green histogram) or treated with vWF (blue histogram) was analyzed by flow cytometry.

Flow cytometric analysis showed that the expression of E-selectin, P-selectin, VCAM1 or ICAM1 on the surface of ECs was not upregulated by treatment of HUVECs with HBSS or vWF (Figure 5) suggesting that upregulation of the adhesion molecules on the surface of ECs was not the reason for the stimulation of the hMSC adhesion.

**Figure 6:**
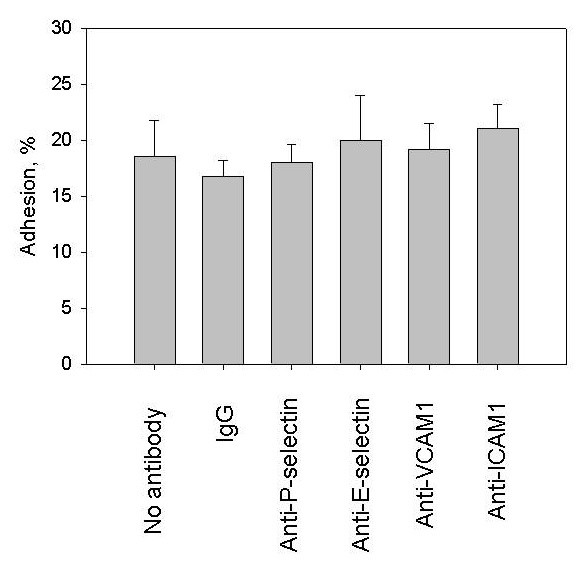
**Treatment of HUVECs with neutralizing antibodies against E-selectin, P-selectin, VCAM1 and ICAM1**. Endothelial cells were treated with 4 μg/ml vWF in HBSS for four hours and exposed to 10 μg/ml matching isotype-control (IgG) or 10 μg/ml neutralizing antibodies against E-selectin, P-selectin, VCAM1 or ICAM1 for 40 minutes before hMSC adhesion assay. Data are shown as mean ± SD of eight independent measurements. Isotype-matching control and neutralizing antibodies against E-selectin, P-selectin, VCAM1 or ICAMP1 had no significant affect on hMSC adhesion to HUVECs treated with vWF (t-test, *P*-value >0.05).

In order to further evaluate the role of adhesion molecules in the regulation of hMSC adhesion to HUVECs treated with vWF ECs were incubated with 10 μg/ml neutralizing antibodies against E-selectin, P-selectin, VCAM1, ICAM1 or isotype-matching control (IgG) for 40 minutes before the adhesion assay (Figure 6). Neutralizing antibodies and isotype-matching control had no effect on hMSC adhesion to HUVECs treated with vWF.

Data of DNA microarrays, flow cytometry and treatment of HUVECs with neutralizing antibodies argue that the stimulation of hMSC adhesion by vWF is not related to cell surface expressions of E-selectin, P-selectin, VCAM1 or ICAM1 in ECs.

### VWF induces the phosphorylation and activation of p38 MAPK and ERK-1,2 in HUVECs

Considering that hMSCs do not directly interact with immobilized vWF and the stimulation of EC adhesiveness for hMSCs is not related to overexpression of adhesion molecules on the endothelial surface, we hypothesized that the effect of vWF on EC adhesiveness is mediated by signal transduction pathways triggered in HUVECs by exposure to vWF.

**Figure 7:**
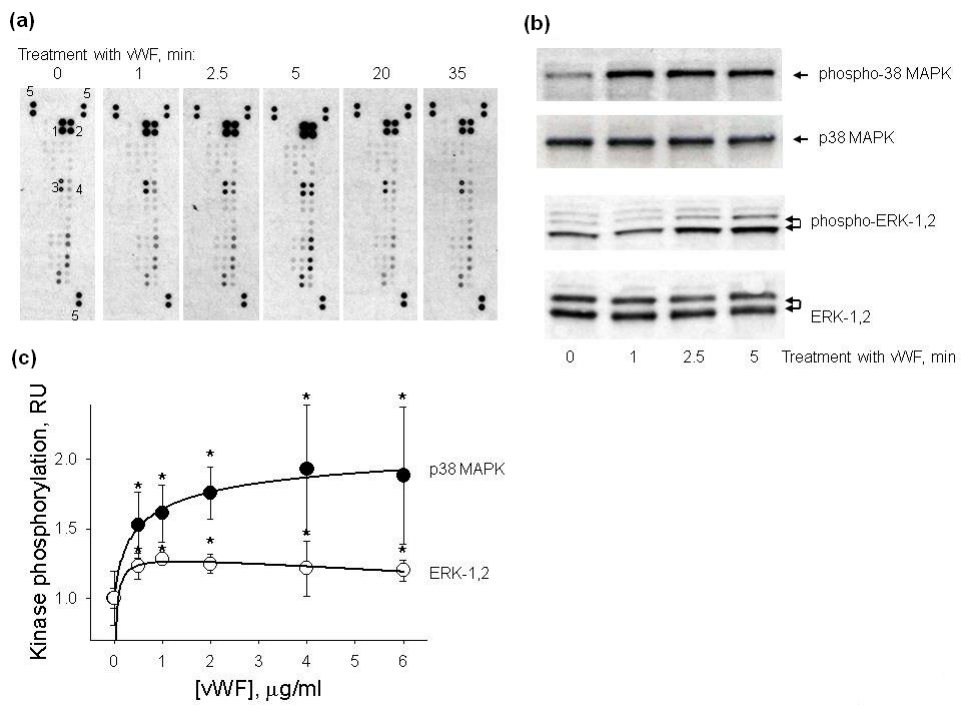
**Phosphorylation of protein kinases in HUVECs treated with vWF**. Protein phosphorylation in HUVECs treated with vWF was analysed using the human phospho-MAPK array, by Western blot and cell-based ELISAs. **(a) **Shows the phosphorylation of eighteen protein kinases in HUVECs treated with 4 μg/ml vWF for 0 to 35 minutes assayed using the human phospho-MAPK array. VWF stimulated the phosphorylation of ERK-2 (spots 1), ERK-1 (spots 2), p38α (spots 3) and p38γ (spots 4). Spots labelled with number 5 are the positive controls used for the normalization of the arrays. **(b) **Shows Western blots of total p38 MAPK and ERK-1,2 and phosphorylated p38 MAPK and ERK-1,2 from lysates of HUVECs treated with 4 μg/ml vWF for 0-5 min. **(c) **Shows the dose-response curves of p38 MAPK (black circle) and ERK-1,2 (white circle) phosphorylation in HUVECs treated with 0 to 6 μg/ml vWF for four hours measured using the cell-based ELISAs. Data are shown as mean ± SD of four independent measurements. Asterisks mark statistically significant changes in comparison with none treated HUVECs (t-test, *P*-value <0.05).

In order to identify signaling pathways involved in the regulation of EC adhesiveness for hMSCs we studied the activation of protein kinases in HUVECs stimulated with vWF. Analysis of protein phosphorylation using the human phospho-MAPK arrays demonstrated that treatment of HUVECs with 4 μg/ml vWF for 0 to 35 minutes resulted in the phosphorylation of p38α MAPK and p38γ MAPK as well as ERK-1 and ERK-2 (Figure 7a). Phosphorylation of p38 MAPK and ERK-1,2 was verified by Western blot analysis (Figure 7b). Quantification of p38 MAPK and ERK-1,2 phosphorylation in HUVECs treated with 0 to 6 μg/ml vWF for four hours was performed using corresponding cell-based ELISAs. Data in Figure 7c show that vWF stimulated the phosphorylation of p38 MAPK and ERK-1,2 in HUVECs in a dose-dependent manner. After treatment of HUVECs for four hours with 6 μg/ml vWF the level of p38 MAPK phosphorylation was increased 1.8-fold (*P*-value <0.05) in comparison with HUVECs in HBSS (Figure 7c). The increase in the level of ERK-1,2 phosphorylation after treatment of HUVECs with vWF was smaller (1.2-fold) but statistically significant (Figure  6c, P-value <0.05).

**Figure 8:**
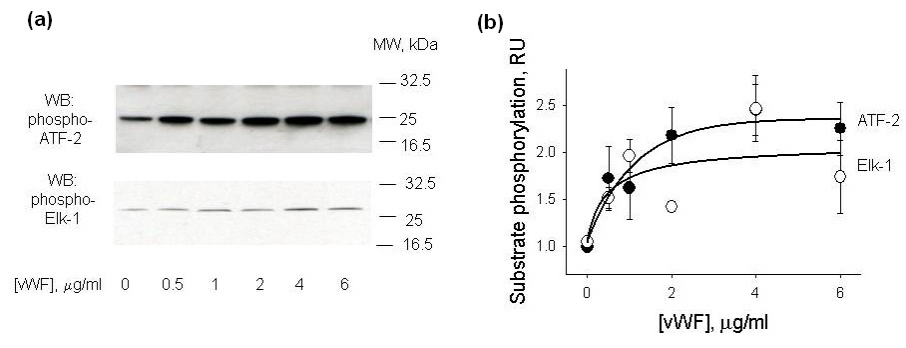
**ERK-1,2 and p38 MAPK activity assays in lysates of HUVECs treated with vWF**. HUVECs were treated with 0 to 6 μg/ml vWF for four hours. Phosphorylated p38 MAPK and ERK-1,2 were immunoprecipitated from HUVEC lysates and their enzymatic activities were analyzed using their specific substrates, ATF-2 and Elk-1, respectively. **(a) **Shows representative Western blots of phospho-ATF-2 and phospho-Elk-1. **(b) **Shows the results of densitometric analysis of Western blots of phosphorylated ATF-2 (black circle) and Elk-1 (white circle). Data are shown as mean ± SD of three independent experiments.

Next, we tested whether the phosphorylation of p38 MAPK and ERK-1,2 in HUVECs treated with vWF stimulates their enzymatic activities. HUVECs were treated with 0 to 6 μg/ml vWF for four hours, phosphorylated forms of p38 MAPK and ERK-1,2 were immunoprecipitated from cell lysates and used to assay their enzymatic activities. Activity of p38 MAPK was measured using recombinant ATF-2 protein. Recombinant Elk-1 protein was employed to test the activity of ERK-1,2. Representative Western blots of phospho-ATF-2 and phospho-Elk-1 are shown in Figure 8a. Densitometric analysis of ATF-2 and Elk-1 phosphorylation is shown in Figure 8b. Enzymatic activities of p38 MAPK and ERK-1,2 were stimulated by vWF in a dose-dependent manner. After the four-hour exposure to 4 μg/ml vWF enzymatic activities of both p38 and ERK-1,2 were increased 2.4-fold (*P*-value <0.05).

Analysis of signal transduction pathways activated in response to treatment of ECs with vWF revealed that vWF stimulates the phosphorylation and activation of p38 MAPK and ERK-1,2 in HUVECs.

### P38 MAPK regulates adhesion of hMSCs to HUVECs activated with vWF

In order to investigate the role of p38 MAPK and ERK-1,2 in the regulation of EC adhesiveness for hMSCs we studied the effects of selective inhibitors of p38 MAPK (SB203580) and ERK-1,2 (PD98059 and U0126) on hMSC adhesion to HUVECs treated with 4 μg/ml vWF for four hours.

**Figure 9:**
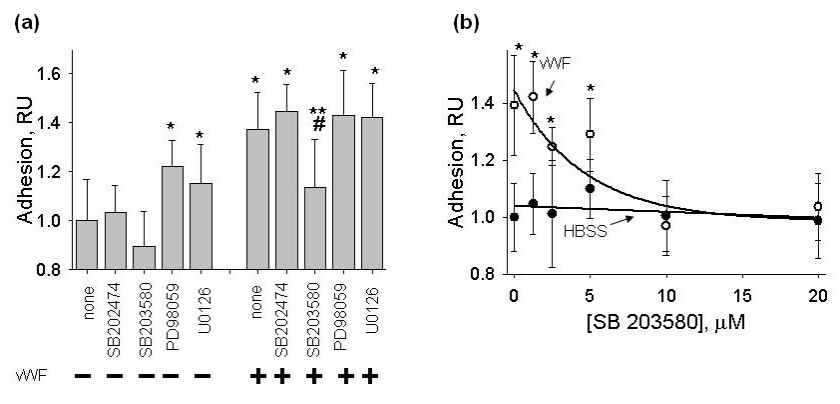
**Effects of p38 MAPK and ERK-1,2 inhibitors on hMSC adhesion to HUVECs treated with vWF**. HUVECs were pre-incubated with p38 MAPK inhibitor SB203580 (10 μM), its inactive analog SB202474 (10 μM) or the inhibitors of ERK-1,2 phosphorylation, PD98059 (10 μM) or U0126 (10 μM), for 45 minutes and treated with 4 μg/ml vWF for four hours in the presence of the protein kinase inhibitors. **(a) **Shows the effects of the protein kinase inhibitors on hMSC adhesion to HUVECs treated with and without vWF. Data are shown as mean ± SD of eight independent measurements. Single asterisks mark statistically significant changes in hMSC adhesion in comparison with HUVECs not treated with vWF (t-test, *P*-value <0.05). Treatment of HUVECs with vWF in the presence of SB203580 resulted in the inhibition of hMSC adhesion in comparison with hMSC adhesion to HUVECs treated with vWF alone (double asterisks, t-test, *P*-value <0.05) or in comparison with hMSC adhesion to HUVECs treated with vWF in the presence of SB202474 (pound key, t-test, *P*-value <0.05). **(b) **Shows the dose-dependent effect of SB203580 (0 to 20 μM) on hMSC adhesion to HUVECs treated with (white circle) and without (black circle) vWF. Data are shown as mean ± SD of eight independent measurements. Asterisks mark data point with statistically significant difference from HUVECs maintained in HBSS.

Inhibitors of ERK-1,2 (PD98059 and U0126, 10 μM) showed a small stimulation of hMSC adhesion (approximately 1.2-fold, *P*-value <0.05) to HUVECs maintained in HBSS and had no effect on hMSC adhesion to HUVECs treated with vWF (Figure 9a). Effect of SB203580 on hMSC adhesion to HUVECs in HBSS was not statistically significant. SB203580, but not its inactive analog SB202474, at 10 μM reduced vWF-induced stimulation of hMSC adhesion to HUVECs by 70% (Figure  9a, P-value <0.05). Data in Figure 9b show that SB203580 inhibited the stimulation of the hMSC adhesion by vWF in a dose-dependent manner (Figure 9b). At 10 to 20 μM SB203580 completely eliminated vWF-induced stimulation of hMSC adhesion to HUVECs (Figure 9b).

**Figure 10:**
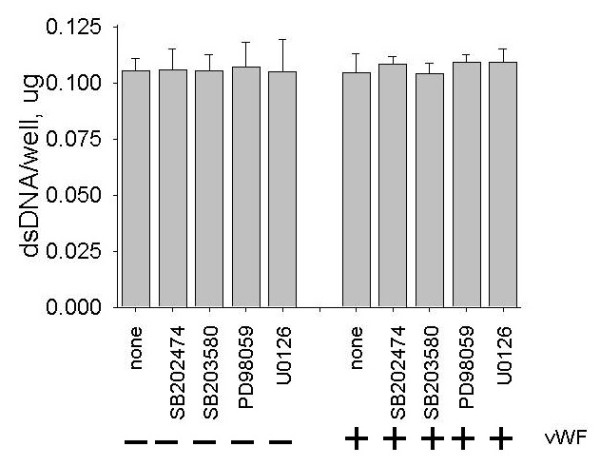
**Effects of protein kinase inhibitors and vWF on a number of endothelial cells in adhesion wells**. HUVECs were pre-incubated with p38 MAPK inhibitor SB203580 (10 μM), its inactive analog SB202474 (10 μM) or the inhibitors of ERK-1,2 phosphorylation, PD98059 (10 μM) or U0126 (10 μM), for 45 minutes and treated with 4 μg/ml vWF for four hours in the presence of the protein kinase inhibitors. The mock adhesion assay was conducted without addition of hMSCs. The number of cells in the wells was assayed using Quanti-iT PicoGreen dsDNA reagent as described in the Materials and Methods. Data are shown as mean ± SD of eight independent measurements. Treatment of HUVECs with protein kinase inhibitors and vWF had no significant effect on a number of endothelial cells in adhesion wells (t-test, *P*-value >0.05).

Visual examination under the microscope showed that the monolayer of HUVECs remains intact at all experimental conditions described above. Cellular DNA content per well was measured to confirm that protein kinase inhibitors and vWF do not affect an endothelial monolayer. Data in Figure 10 show that the same number of cells remains in each well after exposure of HUVECs to protein kinase inhibitors in the absence and presence of vWF.

Data with the inhibitors of protein kinases strongly suggest that the activation of p38 MAPK by vWF in ECs is involved in the regulation of the hMSC adhesion.

## Discussion

Exposure of ECs to inflammatory and other stress factors is a powerful stimulator of cell adhesion. In response to stress, ECs release vWF that binds to EC surface or an extracellular matrix. Interaction of platelets with immobilized vWF triggers signal transduction pathways mediated by GPIb-V-IX complex [8], α_IIb_β_3 _and α_v_β_3 _integrins [10] and leads to platelet adhesion and aggregation [8,10]. In contrast to platelets, the adhesion of leukocytes is mainly regulated by the expression of adhesion molecules on the surface of ECs as the result of *de novo *protein synthesis [9].

MSCs respond poorly to the activation of ECs with inflammatory factors (tumor necrosis factor (TNF)-α or interleukin (IL)-1β) and their adhesion does not correlate with the expression of adhesion molecules on the endothelial surface [6]. At the same time, TNF-α and IL-1β stimulate hMSC adhesion to ECs in the presence of inhibitors of RNA or protein synthesis [6]. The MSC adhesion is also significantly potentiated by other pro-apoptotic agents, like staurosporine, wortmannin and okadaic acid, suggesting that endothelial distress and apoptosis may play a crucial role in the regulation of MSC adhesion to ECs [6]. Adhesion of MSCs to distressed/apoptotic ECs correlates with the secretion of vWF by ECs indicating that vWF may be involved in the regulation of the MSC adhesion [6].

Treatment of ECs with exogenous vWF potentiated the adhesion of MSCs. The presence of vWF in the media during the adhesion assay was not required for the stimulation of the MSC adhesion. It was conceivable that vWF binds to an extracellular matrix or to the surface of endothelial cells, stimulates MSCs and promotes the MSC adhesion. However, the immobilization of vWF on collagen I inhibited MSC adhesion to collagen I coated plates suggesting that MSCs and vWF compete for the binding to collagen I. Flow cytometric analysis revealed the lack of the cell surface expression of α_V_β_3 _integrin and GPIbα, the receptors for vWF, on MSCs. Collectively, these results suggest that vWF does not stimulate MSC adhesiveness, presumably, due to the absence of direct interaction of MSCs with immobilized vWF.

Since MSCs do not adhere to immobilized vWF, we hypothesized that vWF stimulates the MSC adhesion via an activation of ECs. The known mechanism of EC activation assumes the stimulation of *de novo *synthesis and the expression of the adhesion molecules on the endothelial surface. Experiments with ECs treated with vWF showed that vWF affects gene expression in ECs. However, in contrast to the activation of ECs with inflammatory factors, treatment of ECs with vWF did not stimulate gene expression of E-selectin, P-selectin, ICAM1 or VCAM1 and did not upregulate the expression of E-selectin, P-selectin, ICAM1 or VCAM1 on the surface of ECs. These results suggest that the mechanism of EC activation by vWF is different from that described for the activation of ECs with inflammatory factors.

Considering that the activation of ECs with vWF did not rely on the expression of the adhesion molecules we hypothesized that the mechanism of ECs activation is similar to that described for the activation of platelets. It is known that platelet adhesiveness is mediated by binding of vWF with the surface of platelets and activation of signal transduction pathways. The binding of vWF with platelets is largely depend on its interaction with GPIb-V-IX complex [8], α_IIb_β_3 _and α_v_β_3 _integrins [10] and lead to the activation of mitogen-activated protein kinases [8,10] including the activation of ERK-1,2 and p38 MAPK [11,12]. The activation of p38 MAPK in platelets may play a decisive role in the regulation of platelet adhesion and aggregation by vWF [11,12]. Analysis of protein kinase phosphorylation in ECs revealed that treatment with vWF resulted in the phosphorylation and activation of p38 MAPK and ERK-1,2. The inhibition of p38 MAPK, but not ERK-1,2, completely abrogated the stimulatory effect of vWF on EC adhesiveness for MSCs. Thus, studies of signal transduction pathways triggered by vWF in ECs showed that vWF activates ECs via a mechanism similar to that described for the stimulation of platelets. Inhibition of vWF-dependent stimulation of the MSC adhesion by selective inhibitor of p38 MAPK suggested that p38 MAPK plays a crucial role in the modulation of EC adhesiveness for MSCs by vWF.

## Conclusions

VWF is an autocrine/paracrine effector of signal transduction and gene expression in ECs that regulates EC adhesiveness for MSCs via activation of p38 MAPK in ECs.

## Abbreviations

ECs: endothelial cells; HBSS: Hank's balanced salt solution; hMSCs: human mesenchymal stem cells; HUVECs: human umbilical vein endothelial cells; MAPK: mitogen-activated protein kinase; MSCs: mesenchymal stem cells; vWF: von Willebrand factor.

## Competing interests

The authors declare that they have no competing interests.

## Authors' contributions

IAP carried out the experiments and conceived of the study. IAS participated in the interpretation of experimental results, the design of the study and writing of the manuscript. SVD carried out the experiments, conceived of the study and drafted the manuscript. All authors read and approved the final manuscript.

## Supplementary Material

Additional file 1 **Excel file**. List of Affymetrix probe sets with expression values changed more than two-fold (t-test, *P*-value < = 0.05) for HUVECs treated with vWF (4 μg/ml vWF, four hours) vs. HUVECs maintained in HBSS (four hours). Click here for file

Additional file 2 **Excel file**. List of Affymetrix probe sets with expression values changed more than two-fold (t-test, *P*-value < = 0.05) for HUVECs treated with vWF (4 μg/ml vWF, four hours) vs. HUVECs maintained in EGM2 growth media. Click here for file
